# Exploring How Models of Disenfranchised Grief Account for the Lived Experience of SEN Teaching and Support Staff Following a Student Death: An IPA Study

**DOI:** 10.1177/00302228251331399

**Published:** 2025-04-02

**Authors:** Benjamin John Partridge, Rachel L Abbott, Penny J Furness

**Affiliations:** Institute of Social Sciences, 7314 Sheffield Hallam University, Sheffield, United Kingdom of Great Britain and Northern Ireland

**Keywords:** teaching, special educational needs, disenfranchised grief, schools

## Abstract

Special Educational Needs (SEN) describe schooling in the UK for young people with an intellectual disability where mortality is more common than in mainstream school settings. This study explores how well models of disenfranchised grief (Doka, 1989, 2002; Robson & Walter, 2012; Rowling, 1995) account for the experience of staff in SEN school settings. Interviews were conducted with 11 teaching staff, focused upon the death of two children with severe/profound learning difficulties. Interviews were analysed using Interpretive Phenomenological Analysis (IPA; Smith, 2017). Findings demonstrated a lack of disenfranchisement from school-based colleagues, with senior leaders having organisational and financial capacity to actively enfranchise bereaved staff. Within the school context, bereaved staff were the primary bereaved, whereas beyond the school bounds, staff had to renegotiate their rights and entitlements to grieve. Models of disenfranchised grief require some reconceptualisation to fully account for the experience of SEN teaching staff following a student death.

## Introduction

Despite a growing focus in the literature on teacher wellbeing more generally in the UK (Naghieh et al., 2015; Rae et al., 2017) and in other countries (Acton & Glasgow, 2015; Cumming, 2017; Hall-Kenyon et al., 2014), in practice, teachers are rarely discussed as thinking and feeling beings with human emotions (Lazenby, 2006). Despite this, Hargreaves (1998) argues that emotions are at the heart of teaching and inseparable from a teacher’s moral purpose or belief in the role of teaching. While there is this growing focus on wellbeing, there is a limited focus in the literature on grief in education professionals. There are currently very few studies that have focused on teachers experience of bereavement in the classroom (Levkovich & Duvshan, 2021) and very limited literature (Shalev et al., 2024) which explores the disenfranchised grief of teaching staff in a SEN school setting and none in a UK context.

The death of a student in an SEN context poses a range of challenges for education staff, one of which is the unique relationship between staff and students and the potential impact on the recognition of grief in this professional population. Baroness Warnock provided in a foreword to a text by Farrell (2012), which characterises SEN schools in the UK as small communities where both staff and children know everyone and are known to everyone within the community and where teaching happens in small groups or on an individual basis. She also talks of staff working closely and in collegial atmosphere which provides a unique challenge for teaching and support staff following the death of a student when compared to mainstream education settings.

Following a student death, not all staff may feel able to express emotion or may not be recognised as grievers by their peers or others in the wider community. This has been described in the literature as disenfranchised grief defined by Doka (2002, p. 160) as “the grief that persons experience when they incur a loss that is not or cannot be openly acknowledged” and for many this may manifest as an ‘empathic failure’ on the part of the condoler (Neimeyer & Jordan, 2002). Those who experience disenfranchised grief may also be subject to a lack of informal and formal support (Corr, 1998) as a consequence of this lack of recognition.

Doka (1989) initially identified three situations in which grief can be disenfranchised: (a) the relationship is not recognised, (b) the loss is not recognised, or (c) the griever is not recognised. Doka (2002) identified two further situations: (d) the circumstances of the death are not recognised, and (e) the way in which individuals grieve is not recognised (Doka, 2002). The focus for this research is on the first of these, where the relationship between teacher-student is not recognised following a student death. When this is the case and teaching and support staff experience disenfranchisement, their grief often still occurs but in the absence of this recognition of the relationship and their response to the loss may be seen as inappropriate by others, leading to greater difficulties in adapting to the loss (Levkovich & Duvshan, 2021).

Rowling (1995) describes a tripartite model of teacher disenfranchisement following a student death, involving (1) the need to be human, representing the personal response to the loss, (2) the need to control emotional displays and command leadership, reflecting appropriate professional conduct which expressions of grief could undermine, and (3) a duty of care for the young people, thereby maintaining the day to day care and a sense of normality. Providing leadership, particularly in the face of loss, can be overwhelming to teachers, especially when dealing with their own personal reaction (Jellinek & Okoli, 2012).

Rowling’s (1995) tripartite model reflects the personal-professional duality for teaching staff following a student’s death which is also reflected in more modern research by Levkovich and Duvshan (2021). This involves finding a balance between the expression of emotions and controlling those emotions to maintain normality and focus on academic tasks (Hart & Garza, 2013) or support remaining students. This balance is important. Intellectual learning disability does not prevent a child from grieving for their deceased peer and as well as controlling emotion there is a need to normalise grief for young people with SEN (McClean & Guerin, 2019) and after family, education staff are often the next best placed people to do this. Many teachers find it difficult to achieve that balance, to deal with their own emotions and the unpredictability of grief, whilst also managing the children in their care and maintain a sense of control. As a result, teachers will often sideline their own emotions and forefront the care of others (Rowling, 1995). This has been described as a form of self-disenfranchisement (Kauffman, 2002), or as intrapsychic or internalised disenfranchisement (Rowling, 1995), whereby teachers deny their grief experience internally in order to maintain control publicly.

Teachers can experience a sense of loneliness in their grief (Lazenby, 2006). Whilst they provide support for others in the classroom and in the wider school setting, and even outside of the school setting, for example, in supporting parents (Munson & Hunt, 2005; Smith et al., 1991), there is a lack of support for teachers themselves. Lazenby found that few teachers considered their lack of support but, those who had, had expected greater support at an institutional level, perhaps indicative of organisational disenfranchisement. Lazenby does contend that providing support for others can offer an outlet for teachers’ own grief. However, where support is enacted, it often involves senior leaders enabling staff to attend the funeral of a deceased child, for example, which Bennett and Dyehouse (2005) highlight as a way of recognising staff’s humanity, emotion, and their need to grieve. Through these actions of the senior leaders, the grief is both acknowledged and validated, and thus enfranchised.

While generally, the literature is indicative of this organisational disenfranchisement, more recent literature highlights how schools, often co-workers more specifically, are often the most common source of support for grieving teachers (Heller et al., 2013; Shalev et al., 2024). Shalev et al. (2024) in particular found that schools are a good source of support for SEN teaching staff in Israel following the death of a student and staff groups are often characterised by direct discourse, listening and mutual sharing. In many ways this is contrary to the claims of organisational discourse above and instead is more indicative of an enfranchising environment which recognises staff bereavement experiences. In larger staff teams it may be possible that class-based staff may deal with the loss by reaching out to work colleagues or even developing a temporary grief group (Dyregrov et al., 2014), which may support recognition of the loss experience and ultimately enfranchise grief. In the school setting, school nurses, administrators and senior staff or supervisors often act as supportive mechanisms for bereaved teachers (Lazenby, 2006). Considering the wider school population, former teachers of the deceased may also grieve, though there may be little acknowledgement of this (Lazenby, 2006). This is particularly relevant in SEN settings as staff numbers are usually higher, with at least one teacher and multiple teaching assistants per class, leaving a greater trail of potentially affected staff members throughout the school and even in light of Shalev et al.’s (2024) work, it is unclear how past staff associated with the deceased child experience these types of losses.

While there are a number of complexities that exist in terms of rights to grieve, in parts of the UK between 20–39% of schools having some kind of written policy (Holland & McLennan, 2015; Holland & Wilkinson, 2015) with many not considering staff responses to the death of a student. The lack of consistent policy making around bereavement experience for teaching staff in the UK again highlights a degree of organisational disenfranchisement itself with many schools failing to plan for how to manage a student death or recognising teachers as grievers through policy. It is important to highlight that this is UK specific and in other countries such as Denmark (Lytje, 2013, 2017), Norway (Lytje et al., 2021) and Australia (Rowling & Holland, 2000) bereavement planning is more advanced.

Following Doka’s (1989; 2002) seminal work, there has been limited critical examination of disenfranchised grief as a concept in the general populus. However, Robson and Walter (2012) argue that disenfranchised grief is not a binary concept, that is either present or not, but is instead hierarchical. Close family members and primary attachment figures would top the hierarchy; friends, more distant family and secondary relationships would be mid-way; and non-relatives, including professionals such as teaching staff, would be at the bottom of the hierarchy, with the fewest rights to grieve. Robson and Walter (2012) wrote of disenfranchised grief not as a norm but, instead, as a feeling experienced by a mourner whose personal grief reaction exceeds their position in the hierarchy, as perceived generally or by someone above them in the hierarchy Figure 1.

**Figure 1.**
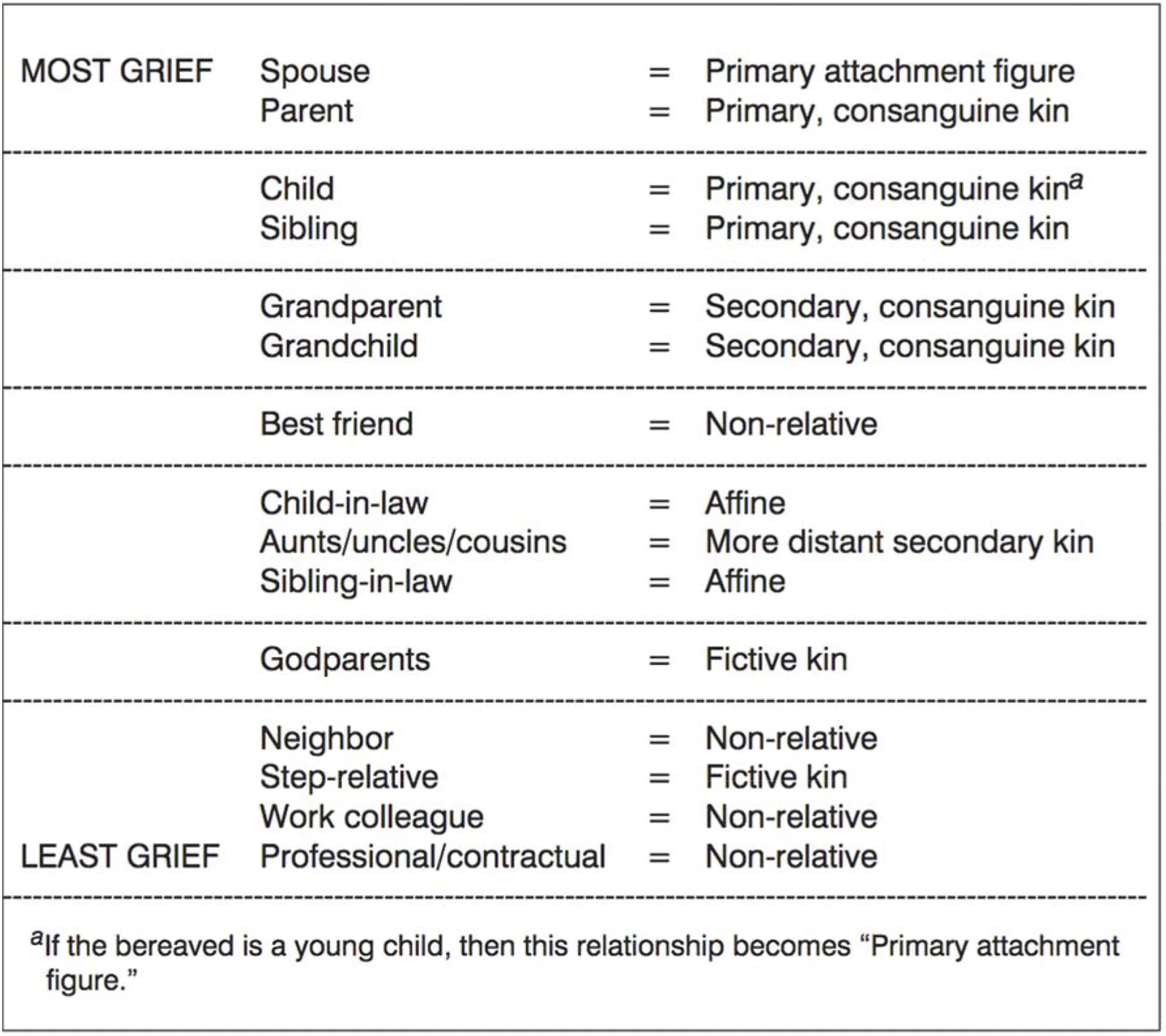
Suggested hierarchy of grief (Robson & Walter, 2012).

Developing Robson and Walter’s earlier critique of disenfranchised grief, the binary assumption can be further questioned when considering the context of the professional griever and the condoler. It is possible that in some social contexts, the professional may experience a lack of rights to grieve openly, resulting in disenfranchised grief, while in other social contexts there is a forum for grief and that grief is recognised. For example, following a student’s death, a teacher may experience disenfranchisement from friends but enfranchisement by a fellow bereaved teacher. It may be problematic, therefore, to assume that all those falling in a ‘professional’ category would necessarily experience disenfranchisement in all social contexts, something that Robson and Walter’s model does not account for.

These influential models of disenfranchised grief provide the theoretical underpinning for the current study.

### Research Context and Aims

The research described here was part of the lead author’s doctoral research programme. Given the limited research focusing on an SEN context, the research took an exploratory approach in investigating the lived bereavement experience of teaching and support staff following a student death. The aims of this paper are to explore the concept of disenfranchised grief, considering how well current models of disenfranchised grief account for the experience of staff in SEN school settings.

## Method

### Design

This study took a qualitative approach to investigating the lived experience of disenfranchisement and enfranchisement for teaching staff following the death of a student in an SEN setting. This research was conducted in 2 schools in the North of England, UK. Semi structured interviews were conducted, and data was analysed using Interpretive Phenomenological Analysis (IPA; Smith et al., 2021). A critical realist approach underpinned the study which assumes a shared objective bereavement experience which can only be understood from the participants subjective positioning.

### Participants

#### Inclusion Criteria for Participation

• Teachers and teaching assistants who were employed by an SEN school and were employed in this role at the time of the student’s death.
• Staff who have experienced the death of a student within the last 3 years but not within the last 6 months.
• Staff who experienced the death of a student in their care; where the student was a member of the class group they regularly supported. ‘Regular support’ was defined as working within a class group at least 3 out of 5 working days or, for part time staff, working all their part time hours within one class group.

11 participants were interviewed: 7 teaching assistants and 4 class teachers. 10 staff identified as female and 1 as male. In SEN schools in the UK class teachers typically lead a class with support from a number of teaching assistants. This may range from 1 or 2 teaching assistants to as many as 10 or more depending on the structure and size of the group but 3–4 is more typical.

Participants Were Clustered round 2 Cases: ‘Owen’, from school A, a nursery aged (in a UK SEN school between the ages of 3–5) child with a severe learning difficulty who died suddenly and unexpectedly following an accident (6 participants);

‘Daisy’ from school B, a secondary school aged child (in a UK SEN school between the ages of 11–18) with a profound and multiple learning difficulties who died suddenly and unexpectedly, due to a degenerative condition (5 participants).

A sample size of below 6 is typical for IPA work due to the depth rather than breadth of analysis (Smith, 2015), however in the current study 6 participants were from school A and 5 from school B, therefore with no more than 6 participants clusters around each case.

### Procedure

A semi structured interview schedule was co-developed by all authors, based on themes in the literature

Participants received information about the study through headteachers (in the UK defined as the person who leads the school) as gatekeepers, following which participants contacted the lead researcher directly to opt into the study and written consent was taken. Participants were interviewed by the lead author, at their place of work during or after the school day. Interviews lasted 57–126 minutes, were audio recorded, transcribed verbatim and then pseudonymised.

### Ethics

Ethical approval was granted by Sheffield Hallam University’s Research Ethics Committee (Reference number: ER6310210) in keeping with the (British Psychological Society, 2021) code of human research ethics (2021).

### Data Analysis

Data was analysed using IPA (Smith et al., 2009, 2021), according to the following steps. The lead author first familiarised and immersed themselves with the data, listening to recordings while reading interview transcripts (Yüksel & Yıldırım, 2015). Each transcript was then coded using exploratory noting and personal experiential themes (PETs) were developed at an idiographic single case level. In line with IPA’s ideographic commitment, comprehensive and detailed notes were made for each participant’s data, focusing upon descriptive, conceptual, and linguistic comments. Once PETs were generated, group experiential themes (GETs) were developed around school A and school B which involved undertaking a cross-case analysis of the two case clusters separately. A further layer of analysis, involving a super-cross-case analysis was conducted, generating themes across the two case clusters, involving all 11 participants in the study. Cross and super-cross-case analysis involved exploring convergent and divergent points across all cases, balancing these findings with the ideographic findings (Smith, 2015). Initial coding was led by the first author, but all authors met to consider and discuss the lead author’s emerging ideas at PET, GET and cross-case-analysis stages, and in developing and agreeing the final themes.

## Findings

### Theme 1: Enfranchising Actions of Colleagues and Senior Leaders

Across schools A and B, all participants described experiencing support from several sources but most notably from colleagues in class and the school senior leaders. Colleagues in class shared directly in the experiences and appreciated the sense of sameness. Senior staff acted as gatekeepers to both financial and organisational resource and capacity, and also had a key role in establishing the culture within the school. This school culture provided the context in which bereavement experiences were expressed. In school A in particular, there was a pre-established supportive and enfranchising culture within the school which positively affected the way in which staff experienced the loss.

### Shared Experiences with Colleagues

Class staff reported a good sense of social support from peers, particularly from within the class team. It was evident that staff turned inwards to the team for support, rather than outwards to peers in the wider school, due to the sense of sameness of experiences that staff in the class team share.

Berni talks about the day in which she was notified of the loss. Here staff made a decision to stay together as a class, rather than integrate with the wider school.

“On the day, I’m not sure. Like I say, we kind of kept in our classroom, I think, mostly that day, if I remember rightly. We didn’t really want to go down to the staff room, we just stayed together. Do you know what I mean? So, that day, I don’t really know that I encountered many other people that were sort of outside our team.” (Berni, School B)

Berni talked about not doing some of the things that she would normally do, such as go to the staff room, opting instead to stay with the rest of her team. Here she made a distinction between the class team and the rest of the school, reflected in the choice of physical space. She stayed in the classroom, turning inward for support to those who shared in her experiences. She used the word ‘we’ which indicates a collective decision, one that was made by the team as a whole, and her language reflected that a sense of unity. The team as a whole chose to stay together rather than see people from outside the team in the wider school or visit parts of the school where this might be the case. Those within the class team had the same experience, were able to empathise and understand the experience, something which those in the wider school may have been unable to do. The class team in having this shared experience potentially provide a place of solace and support.

Much like Berni, Andrew described the period of time after being notified of the loss and the supportive nature of the team.

“we all sat there for the full afternoon we were told we could have the entire afternoon out if we wanted to go home at that point we could but we all stayed to support each other through it erm and we just tried to say as many positive things we could think of” (Andrew, School A)

While staff were given the option to go home or have time away from school or the class following the notification of the loss, Andrew described how everyone chose to stay together and provide a supportive environment for each other. What Andrew described here is a collective mourning of the loss and a shared sense of grief. The sense of shared experience following the notification of the loss was important in creating a supportive and enfranchising environment in which it was understood that everyone had experienced loss and that it needed to be marked and expressed.

For Andrea, the sense of bondedness and being around others with the same shared experience was important and almost therapeutic.

“I think that was just the therapy of having people around you who knew what everybody else was going through but not necessarily talking about it 24 hours a day. We were there and we knew what happened, we knew what we went through” (Andrea, School A)

Andrea found some sense of support in working with people who understood her experiences and had shared in these; a sense of support which was almost therapeutic in nature and beneficial to her. There was a shared experience between her and other members of the team. If the school were to bring in someone from outside the team, they would lack the same, important shared experiences. Furthermore, Andrea didn’t need to talk about the events surrounding Owen’s death every day, instead just knowing she had this shared experience with the other members of her team was enough to feel this sense of support and enfranchisement. This was often an unspoken understanding, a silent support for her.

Andrea continued this discussion by talking about the peer support she received following the loss and the importance of this shared understanding and experience that her peers had of Owen.

“peer support I would say, just being with people who feel the same and who felt the same about him and who experienced him in the same way as I did, you know? Like I said, we did fight over him like that, but there were other people there who loved him the same way, you know? I would say he was mine or whatever but, they all felt the same. Just to know we’re all going through the same stuff and we could chat about it.” (Andrea, School A)

In having a shared understanding and shared experiences with her peers, Andrea felt supported. This support may have been verbalised but was also unspoken and did not need to be articulated for her to know that it was there. Andrea experienced an enfranchising sense of solidarity through her peers, and the sense of sameness of experience had benefits in being able to talk about what had happened.

### Facilitation of Financial and Organisational Capacity and Resource by Senior Staff

Across School A and B, participants experienced several practical actions that they felt recognised their experience of loss and their rights to grieve. Notably, time and space were two of the most important resources that class staff reported as being important within the loss. Andrea spoke of the unpressured time they were ‘allowed’ as a team without the presence of children in the room and the physical space provided when senior leaders removed children and dispersed them into different classes for the afternoon.

“they’ve never said, “Oh, you have to be back because… We were given the time to grieve and to be sad .... I think that day when they took the kids off us and they said, “It’s not your problem, that’s what happens,” and you just deal with yourself in terms of you’ve got the right to be upset, don’t worry about the kids, they are sorted, they are safe, they are happy, that is your time to get that information.” (Andrea, School A)

Andrea talked about not having children present and having responsibility removed for anyone other than themselves and one another as a team. Doing this naturally allowed for time and space and for the team to collectively mourn the loss. In experiencing this removal of responsibility, Andrea described a scenario where her needs and the needs of her fellow team members were put first and prioritised by senior staff who had the organisational capacity and resource to make such decisions.

Andrea also indicated here that she felt senior leaders recognised her and other team members’ right to be upset and anticipated an emotional reaction, providing a rationale for the time and space they were granted. Senior leaders’ reassurances about the children in the group, that they were happy and safe, further served to remove from Andrea any additional distress or concern for the welfare of children. Critically, Andrea and her team members had their personal needs recognised, rather than merely being helped as professionals to support the children they cared for.

In addition to time and space, Brenda talked about being able to make decisions and have autonomy. This autonomy could be thought about as a conscious lack of action from senior leaders, enabling class staff to take control.

“We wanted to stay together as-We were offered an option, if we all wanted time out… we needed to stay together because we all felt the same.” (Brenda, School B)

Autonomy over decision making *by* the class, *for* the class, was empowering. Brenda and other members of her team were able to dictate their own path through the loss within certain parameters, rather than having decisions forced upon them, and, therefore, experienced a degree of control.

I: “is there anything you think could have been done better?”

B: “No, because they are caring. No, I don’t think so, because they were forever watching us and making sure we were alright and popping in, to make sure things were okay. No, I think they did well.” (Brenda, School B)

Brenda also talked about feeling supported by senior leaders; she saw them as having an overview of the situation and the class, while at the same time enabling staff to act autonomously, both in decision-making as exemplified above and the day to day running of the class following the loss. Brenda saw senior leaders’ approach as caring and the support she received as appropriate.

Andrew described the arrangements made around Owen’s funeral. He talked about the experience of attending the funeral and being able to travel to the funeral with his colleagues, again being allowed to do this by senior staff in school.

“there was that many of us going that they put two school two minibuses on for us at school so we were told to come in as normal but we were all allowed time in class 1 where we were all sat together we didn’t go into class with the children” (Andrew, School A)

Crucially, Andrew was facilitated by senior staff to attend the funeral and, by making use of their financial resource to supply a minibus for staff in which staff members were able to stay together and support each other, they recognised the shared experience and the shared grief of class staff and the importance of their being able to connect with one another. Interpersonal relationships were acknowledged and encouraged by the collective travelling, and participants were able to act as a source of support for each other, which recognised staff’s rights to grieve their loss. As above, Andrew used the word ‘allow’ when describing his experience, indicating the privileged position he and his colleagues appreciated through the support they received, rather than viewing it as an expectation or a right.

### Enfranchising and Supportive Culture and Ethos

As well as recognising the organisational and financial resources offered, participants in school A described a culture of mutual respect and collaboration. Alice talked about the way in which she collaborated with Bill, the school’s head teacher, of not ‘being managed’ or ‘steamrolled over’.

“And, again, asking me, and giving me the option to tell the team, and giving me the option to read the poem. You know, asking me about things, and not just steamrollering over me, and managing it.

We were all very much in it together, I think, as well, in that area. It’s everything. You know, it’s discussing things and asking questions and, “Do you want to do this? You don’t have to. No pressure.” So yes. I think both of them have been tremendously supportive.” (Alice, School A)

In this scenario, instead of feeling ‘pushed aside’ within the loss, Alice felt valued by senior staff, and having her relationship with her team as well as with Owen recognised. Alice spoke of being ‘in it together’, adding to the sense of collaboration and mutual respect Alice experienced.

Collaboration was also important for Anna and Andrea. They described experiencing a culture of open communication between themselves and senior leaders.

“they kind of didn’t wrap us in cotton wool but they gave us a lovely little nestling embrace and they said they were very much open about the fact that tell us what you need we can facilitate that and that was really really good” (Anna, School A)

“I think I would… Good. Good in terms of we were given time for the funeral, we were given time in the day to grieve and process the information and I think the support was there with their door open all the time.” (Andrea, School A)

Anna’s use of the metaphor ‘nestling embrace’ here indicates that the support she and her team received felt appropriate to her, rather than ‘wrapping us in cotton wool,’ which would have meant being swaddled, over-supported and unnecessarily shielded from the experience. Within the context of the loss this embedded feeling of support was valuable in recognising and enfranchising the loss. Anna reported no limits on the support that she felt senior leaders were willing to offer her, which she clearly appreciated. For Andrea the on-going nature of this support was very positive. She described the open door being there ‘all the time’, indicating that she did not see this support as just situational, offered in response to the notification of the loss, but that she experienced an on-going sense of support. For Andrea this felt empowering without being overpowered: the door being open, but nothing forced and another example of her experience of loss being recognised.

Anna felt that the support she was offered by senior leaders was genuine and heartfelt.

“or just knowing that management isn’t just some number cruncher just thinking of the bottom line” (Anna, School A)

Anna’s statement above helps to provide a better understanding both of the way she viewed senior leaders and of how she felt she was viewed by them. She saw them as acting in a way that was considered and heartfelt, but also saw herself as valued, respected and recognised in their eyes.

In school A, Anna talked about the power of this genuine support, identifying that, for her, the offer of support in this case was as good as having the support actioned. The offer alone and knowing that she could take senior leaders up on it was powerful. She saw this offer of support as empowering and it allowed her to continue to work, be with the rest of her team and manage her own loss experience.

“they were really really understanding and it was they made it it was as good an experience not that it was good they tried to make it as comfortable for all of us as they could and you could feel that you could really feel that if you went to Bill one day and said I can't do today he would have said go home its fine and I think because of they that they didn’t have any of us go off ill erm that none of us needed that compassionate leave or stress or anything like that so I think that was really really good” (Anna, School A)

“having that knowing that you could talk or knowing that you could have that you probably could have time off if you needed it was almost as good as having the time off I don’t know if that makes sense it's it’s the thought of being able to do something means that you are kind of able to cope with it” (Anna, School A)

Ultimately Anna felt that her experience was both recognised and respected by senior leaders. She talked of ‘feeling’ this sense of support, that the support that Anna senses was unspoken by senior leaders. That sense of support may have reflected her experience of actions in this particular situation as well as being deeply embedded within the school culture. This sort of culture and ethos is most likely to be pre-existing rather than reactive as this unspoken sense of support is difficult to establish in the short term. For Anna, that unspoken culture of support was pivotal in being able to cope with her experience.

### Theme 2: Navigating Rights and Entitlements as Professionals outside the School Context

Within the boundaries of the school setting, these teaching and support staff were the primary bereaved and experienced the ability to grieve the loss openly and have their bereavement enfranchised by colleagues and senior staff. However, outside of this school setting, teaching and support staff had to renegotiate their role in relation to the child’s primary attachment figures, the child’s parents, which affected the way they saw their position in the wider social landscape or ‘geography’ of the loss. Teaching staff often positioned parents as the primary bereaved and in comparison, they saw themselves as playing a more peripheral role, particularly when attending the funeral or burial which in an SEN school context may be some of the only times staff see family members following a student death. While some participants had clearer, more boundaried ideas about their role, others were less certain and boundaried in their attempt to navigate this landscape for the first time.

Ali talked about her experience of attending the funeral, observing and interacting with parents in this setting.

“you never you know to say to someone do you in that situation you know and you are sad yourself and you think god they must be sadder than me” (Ali, School A)

Ali compared her position with that of Owen’s parents, who ‘must be sadder than me’. This statement reflected how Ali saw her experience of loss and position as lower on the hierarchy than other key figures in the child’s life. For Ali, being a parent meant that you have the right to a stronger, more intense grief response, you have the right to feel sadder, whereas being a professional in this context meant that you would (and perhaps, should) not feel the same as a parent would. Ali attempted to make sense of her position at the funeral through this linguistic work and, like others, this perception of an unspoken grief hierarchy dictated her expected experiences and rights relating to loss.

Andrea also talked of being at the funeral and the burial, and discussed her role and rights here.

“For me, it’s a family moment. Even though we were a school family… Yes, they had more right to be there than we did, that’s how I feel” (Andrea, School A)

Andrea was very clear about how she saw her place within the setting of the burial and at the graveside. She thought of this as a time for family, for Owen’s parents, and that his family had more right to be at the graveside than she or her colleagues did. However, in her use of the phrase ‘even though we were a school family,’ Andrea indicated that she saw her team as a type of family and that, being there with the other members of her team, was the professional equivalent of such a personal experience. In making reference to the idea of a ‘school family’ she attempted to categorise herself and her peers in a way that would give them more rights in relation to the loss than a typical ‘professional’. However, despite this, she didn’t attribute to herself the same rights at the funeral as Owen’s biological family. In terms of a hierarchy, she assigned herself rights which were more than a typical professional or tertiary relationship but not as great as those of the primary caregivers and family. Here, Andrea’s use of language was nuanced and reflected careful social positioning, whereby she was making sense, understanding and negotiating her rights and entitlements in relation to others around her. She positioned herself against the family in this socially setting in a way which was littered with hierarchical complexities.

Andrea went on to describe her experiences of burials previously which helps to provide a context for her thinking and further exemplify the way in which she thought about her role within the burial.

“The very close family will stay by the grave, you will just go and say condolences and leave, that’s how I felt. At that point they need to be left, so that culturally was my bit. The same happens, you say condolences, you offer comfort, but you move, you don’t just hang around, you leave the family to have… That’s what I meant by intimate moment, that’s their moment.” (Andrea, School A)

“Rather than everybody staring at you and watch you go back, that’s the moment when I would probably say, “That’s it.” You say, “Goodbye, you’re going to the ground.” I think condolences, leave, and let the family intimately finalise their bit, if you know what I mean?” (Andrea, School A)

Andrea’s role at the funeral was more transient. While she felt able to approach the graveside and have time to say her goodbye, to give her condolences to the family, for her, the burial is a time for the parents, and the parents were the grievers here to whom condolences are offered. To some degree this may be culturally determined by her prior experiences, but this may also be socially determined based on the perceived rights and entitlements that she holds in relation to her professional role and relationship to Owen following the loss. Andrea talked about this as an intimate moment and through her discussion she perhaps implied that she saw her presence at the funeral as more of a privilege than a right. The rights here are reserved for family and, as a professional, she was privileged to be able to attend the burial.

While her experience was similar to Andrea’s, Alice appeared less sure about what was appropriate or inappropriate at the burial. She looked to others for support in making sense of what could and could not do at the burial.

“I remember looking at someone and saying, “Do you think we could go and say goodbye?” and she said, “Yes.” So, I did, but I don’t know why I felt like I needed to ask for approval really.

Because I’m not family, I think. I don’t know. I don’t know…but, yes.

Again, I was like, “Yes. I’m glad about that, too,” because if you can go… and you don’t feel like you’ve got to talk out loud. You can be there, and you can say your own goodbyes in your head, and blow his bubbles.” (Alice, School A)

Where Alice’s experience differed from Andrea’s was in her attempts to negotiate her role and the associated rights and entitlements around this role. She did not have a clear or defined sense of the appropriateness and inappropriateness of her actions as a professional at the funeral or burial. She looked to others for support in understanding what would be appropriate and possible as a professional. She talked about not knowing if she could say goodbye or approach the graveside because she was not family and indicated both what she feels the rights of family are and her initial thoughts about her own. In falling into a tertiary or professional category, she considered hers a less central role in the funeral or burial. She talked of saying goodbyes in her head rather than saying these out loud, with her role as more observational and passive than active.

## Discussion

Doka’s (1989; 2002) model of disenfranchisement is helpful in understanding the disenfranchised nature of the grief experienced by these SEN teaching and support staff. Doka’s model suggests that where rights to grieve are not recognised, disenfranchisement occurs. In the current study, particularly in School A, colleagues and senior staff recognised class staffs’ rights to grieve and were well positioned to do so, having organisational and financial capacity to not only recognise their grief, but actively enfranchise it. This was done by providing time and space and resources to attend the funeral as well as a school culture and ethos where feelings of support was inherent and embedded. If Neimeyer and Jordan (2002) talk of disenfranchisement as an ‘empathic failure’ on the part of the condoler, in the current study, senior leaders may be described as both empathic and as actively enfranchising staff by both their reactive and proactive actions.

In light of Rowling’s (1995) tripartite model of teacher disenfranchisement, the actively enfranchising actions of senior staff both recognised staff as grievers and also recognised their need to express human emotion. The collectivised nature of the loss and the removal of professional responsibilities, involving the redeployment of students which in turn alleviated the duty of care for other children and the unpressured time to grieve like in the case of Andrea, provided a forum for teaching and support staff to explore their grief without having to be ‘in control’, and thus avoiding the potential for intrapsychic or internalised disenfranchisement (Rowling, 1995). This removal of responsibilities and ultimate enfranchisement may well be dependent on a number of factors such as staff resources, senior staff attitudes and previous experience of bereavement in schools and senior staff empathic capacity and focus on staff wellbeing. While this was the case in school A in particular, this may not be a norm for all schools.

The work of Robson and Walter (2012) is designed to understand the notion of disenfranchisement in the general grief context, however when considered from the lived bereavement perspective of SEN teaching and support staff, important findings emerged that might help to develop or reconceptualise Robson and Walter’s model. SEN teaching staff have a unique working environment, class and school structures and relationships with children and families. Findings from this study demonstrate how SEN staff experienced their rights and entitlements within the bounds of the school setting and how these differed at events in the wider social setting, for example when attending events such as the funeral or burial.

Robson and Walter (2012) wrote about hierarchies of grief, demonstrating that in every loss some people are attributed more rights to grieve than others. This research proposes the notion of hierarchies of grief to be a useful one in understanding the experience of disenfranchisement but do differ within different social contexts. This then extends Robson and Walter’s ideas in reimagining their model through the eyes of bereaved professionals. Findings show how in the ‘out of school’ context, teaching staff fall towards the bottom of the hierarchy as explained by Robson and Walter, becoming some of the least affected in comparison to family members and friends. The data supports this notion that education staff in this context understand their relative position in this social landscape by making these comparisons, positioning themselves as having less entitlement to grieve the loss than these primary family members. Conversely, within the social context of the school, class staff become the most or ‘primary’ affected people, having the greatest rights to grieve following the loss, as family and friends do not form a central part of this social environment. This is an important contribution to understanding the social positioning of professionals following loss and how the social context can affect their rights to grieve but how education staff can experience this paradoxical state of being in some ways both most and least affected by the losses they experience.

Figure 2 below, provides a visual representation of the researcher’s suggestion for how Robson and Walter’s (2012) model could be adapted to account for the contextual differences, proposing a secondary hierarchical structure within their original structure. It is imperative to point out at this stage that while the notion of secondary hierarchies is based on the data from this study, the actual proposed model shown below is not based on empirical data from this study and the current data is not specific enough to make suggestions about the placement of key figures within this secondary hierarchy. Below is a suggestion of how the existing hierarchical model might apply in this scenario and where different people within the school community may fall on this hierarchy to illustrate the thinking. These ideas would need to be empirically tested, and future research could explore the rigour and validity of such proposals.

**Figure 2.**
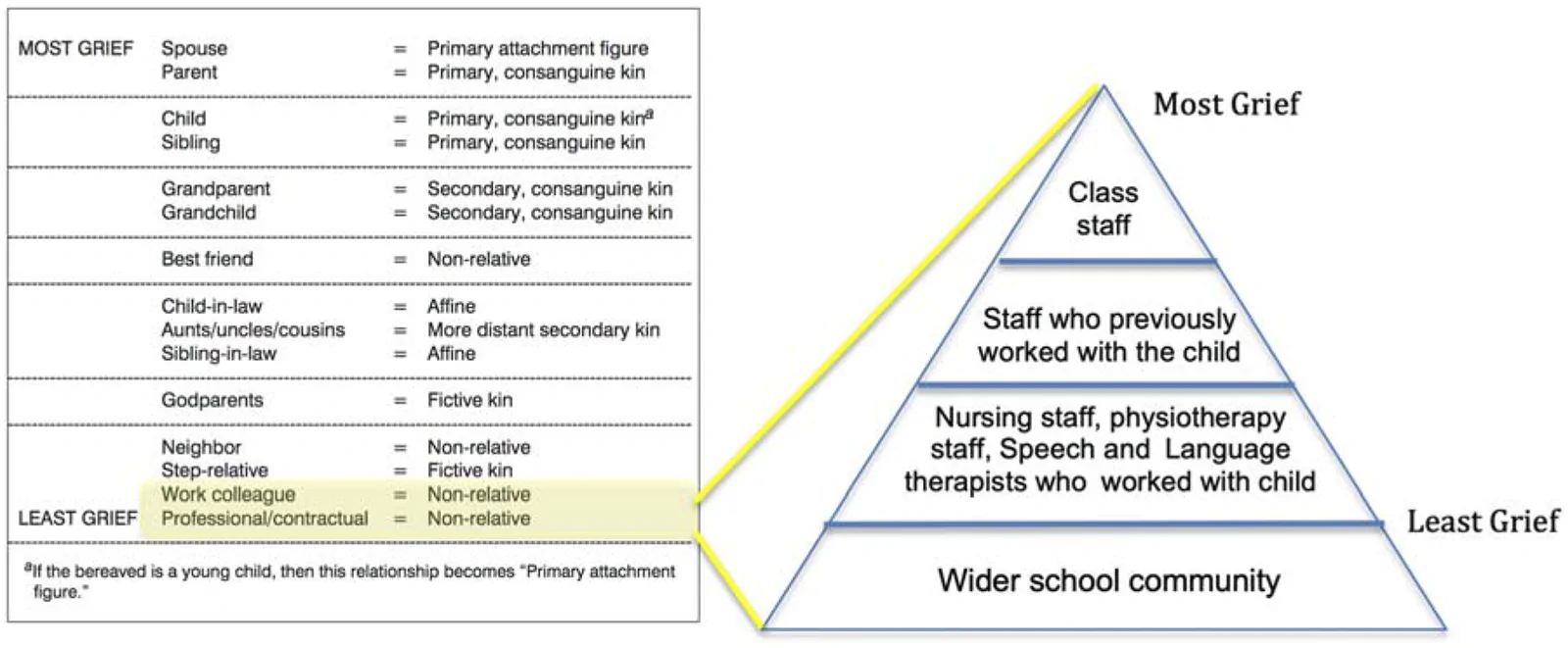
A reconceptualisation of Robson and Walter’s (2012) model.

Overall, Robson and Walter’s model is a helpful model in understanding (dis)enfranchisement in SEN teaching professionals by understanding grief as heirarchial but the model should also consider the social context of the bereaved and the condoller to better capture and understand the experience of professionals.

### Implications and Contributions

Findings from the current study highlight the way in which education professionals in SEN settings experience differential rights and entitlements to grieve in differing social contexts. While the current study focuses on the ‘within school’ and ‘out of school’ contexts of teaching and support staff, it highlights the importance of social context in understanding disenfranchised grief, subjective feelings of rights to grieve and hierarchical attributions of rights to grieve. Future disenfranchised grief research should consider and explore the role of social context.

In terms of the generalisability of the current findings outside the SEN setting there are some key differences between SEN and mainstream school settings. Research demonstrates that the organisational structure of SEN schools and number of education professionals per class (Farrell, 2012) is different to that of mainstream schools and for many student deaths the cause of death may be linked to their learning disability which may lead to further differentiate the experience. In addition SEN staff have a professional closeness at all ages which might typically only be seen in mainstream settings in younger children (Hargreaves, 1998), often requiring greater degrees of touch and physical contact to fulfill all forms of care needs (Hewett, 2007), including personal and intimate care. With this in mind, the nature of the relationship between staff and students is also different. However, despite these differences, the key take home from the SEN setting, applicable to mainstream settings is that senior staff are in a privileged position of having financial and organisational capacity to enfranchise staff in many ways and this transcends who the school caters for in terms of age range or ability.

In terms of the ability of senior staff to enfranchise, this supports some of the existing research around SEN schools (Shalev et al., 2024) and co-workers (Heller et al., 2013) as key sources of support. Going forward there are some key considerations for how to improve and formalise this support. While some of these actions can be reactively implemented, careful planning and preparation is integral for the effective implementation of both short term and longer term actions to enfranchise staff. Many of these more reactive considerations such as allowing time and space or providing financial support to faciliate staff to collectively attend the funeral of a deceased student would still benefit from planning and preparation either to ensure there are suitable finances or to ensure that these actions are applied consistently across all events in order to treat staff fairly. Furthermore, senior staff should carefully consider how they pre emptively embed a supportive culture and ethos as part of the process of planning and preparing for student death in SEN contexts.

Future research in this area should focus on exploring the prevelance and content of bereavement planning and policy in UK SEN schools. There is no clear picture of bereavement planning in the UK, however even if it is never used, research demonstrates how the presence of bereavement policy demonstrates that an organisation take work related bereavement issues seriously (Tehan & Thompson, 2013). In institutions such as SEN schools where staff have the potential to encounter multiple losses, this demonstration of commitment to staff wellbeing and is an important step in creating an enfranchising environment by formally recognising staff as potential grievers. In the current study, some participants had experienced multiple deaths over their career and commonality of bereavement experiences alone appeared to have little impact on the schools response, instead, school responses are individual and driven by school leadership at a whole school level and driven by a collective response at the class based level. In order to see significant changes in response to bereavement, in the absence of national policy and initiatives which educate education about bereavement and promote the recognition of staff as grievers, school based policy is a positive step forward in tackling disenfranchisement.

## Concluding remarks

Current models of disenfranchised grief go some way to explaining the experience of SEN teaching and support staff. However, given the intent of these models to explain disenfranchised grief in a generlised context, the models may be too broad to explain grief in SEN schools and there may be secondary heirarchial structures in place within specific social contexts such as within school settings that might attribute differential rights to grieve beyond the wider social landscape of the loss. Furthermore, lived experience of participants in the current study highlight enfranchising experiences from senior staff and colleagues which recognise participants rights to grieve in these tertiary relationships. In the wider social context of the loss, at key events such as the funeral and burial, educational professionals may have less well defined roles. Education staff may need to negotatiate their rights and entitlements at these events and will often position themselves against primary kin as a way of understand what these rights and entitlements are. These findings may apply to a wide range of professional education based workplace settings and have potential for application beyond SEN school settings as discussed above. Furthermore, future research should focus on exploring organisational disenfranchisement and the role of policy in recognising staff as grivers and establising a culture and ethos of enfranchisement in UK education.
